# Caseous calcification of the mitral annulus: case report

**DOI:** 10.1590/S1679-45082013000300019

**Published:** 2013

**Authors:** Lucas Arraes de França, Ana Clara Tude Rodrigues, Marcelo Luiz Campos Vieira, Wércules Antônio Alves de Oliveira, Rudyney Eduardo Uchôa de Azevedo, Adriana Cordovil, Edgar Bezerra de Lira-Filho, Claudio Henrique Fischer, Samira Saady Morhy

**Affiliations:** 1 Hospital Israelita Albert Einstein, São Paulo, SP, Brazil.

**Keywords:** Mitral valve, Calcinosis, Mitral valve insufficiency, Echochardography/methods, Case reports

## Abstract

We present a rare case of probable caseous calcification of the mitral. This pathology is more frequently detected in asymptomatic women older than 70 years. To recognize this image is important because echocardiography is the easiest way to elucidate this diagnosis, and more importantly because this structure could be easily misdiagnosed as tumors, thrombus and vegetations, which are much more common. Normally, it has a benign evolution, and the correct diagnosis is crucial to avoid unnecessary surgical interventions.

## RESUMO

Relatamos um caso raro de provável calcificação caseosa do anel mitral. Essa patologia é mais frequentemente encontrada em mulheres assintomáticas e com idade acima de 70 anos. O reconhecimento dessa imagem é importante, primeiramente devido à ecocardiografia ser a forma mais fácil para elucidação diagnóstica e também por ela ser comumente confundida com outras massas, como tumores, trombos e vegetações, entidades mais comuns. Habitualmente, apresenta evolução benigna e seu correto diagnóstico é fundamental para evitar intervenções cirúrgicas desnecessárias.

## INTRODUCTION

Caseous calcification of the mitral annulus is rare and accounts for 0.5 to 1% of calcifications of the mitral annulus. It often occurs in elderly women (older than 70 years) and is considered an important differential diagnosis for heart tumors, thrombus or vegetations^(1,2)^.

## CASE REPORT

An 83-year-old woman (weight, 80kg; height, 160cm) was referred to echocardiography service of the *Hospital Israelita Albert Einstein* in São Paulo (SP) to undergo a transesophageal echocardiogram in order to investigate the diagnosis of an intracardiac mass visualized through a transthoracic echocardiography in another service. Her clinical history included systemic arterial hypertension, dyslipidemia and obesity degree I (body mass index – BMI: 31.2kg/m^2^). She reported effort dyspnea, but refused to have other cardiovascular symptoms.

Following the service routine the patient was submitted to transthoracic echocardiography before the transesophageal study. The transthoracic echocardiogram showed important enlargement of both atria and mild increase in the thickness of the heart muscle. Global systolic function of left ventricle was preserved (ejection fraction; 0.67), and no changes in segmental myocardial contractility were seen. The analysis of diastolic function showed diastolic standard of restrictive left ventricular filling (relation E/A >2, with relation E/E'>15). The aortic valve was thickened without restriction to its opening and had a mild reflux. Mitral valve was slightly thickened with normal opening and had mild to moderate regurgitation. Tricuspid regurgitation was moderated and enabled to estimate maximum systolic pressure of pulmonary artery at 106mmHg. Ascending aorta had a mild ecstasy in tubular portion measuring 3.8cm. Pericardium showed normal echocardiographic aspect.

**Figure 1:**
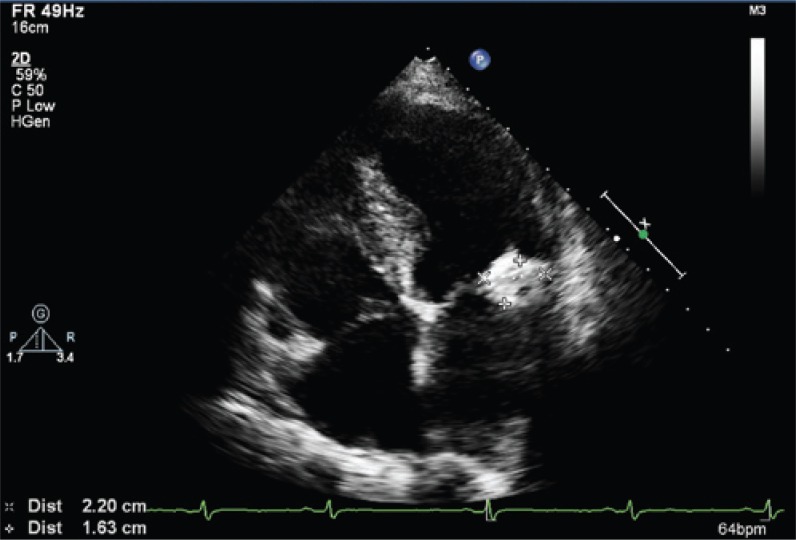
Apical 4-chambers view showing the mass located in posterior mitral valve annulus

In the transthoracic study was possible to observe a mass of heterogeneous content with calcification points, regular edges and more echolucent central portion placed in the posterior mitral valve annulus (coarse calcification of annulus). The mass did not determine any restriction to the opening of mitral valve cusps (Figure 1).

**Figure 2:**
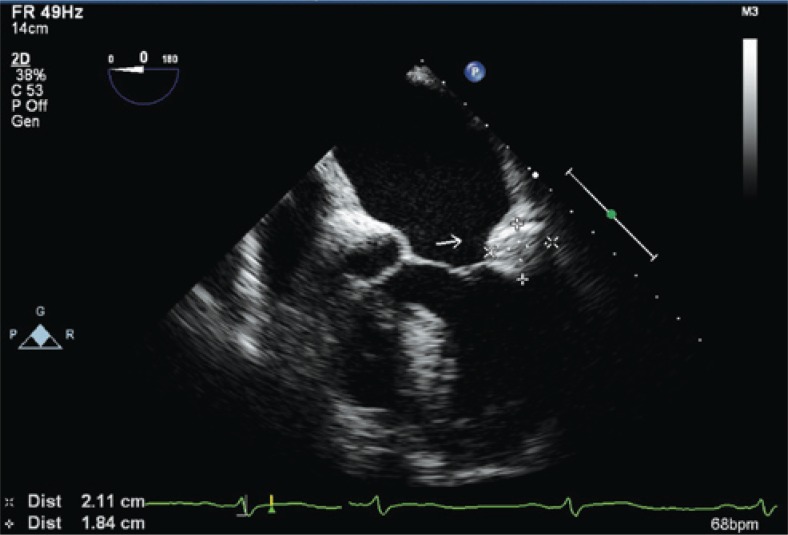
Transesophageal echocardiogram showing a heterogeneous mass and calcifications points located in posterior mitral valve annulus

**Figure 3:**
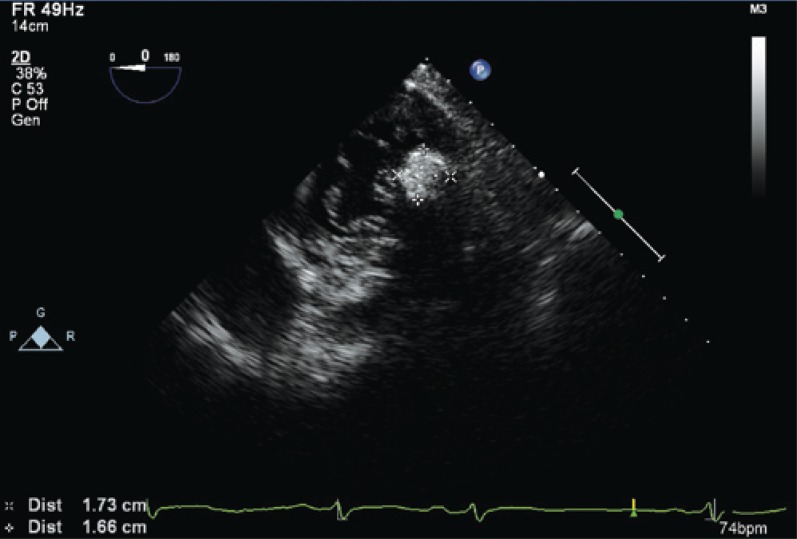
Transgastric view by transesophageal echocardiogram in mitral valve level showing the same mass located in the posterior mitral valve annulus close to P2 segment

The patient was sedated with midazolam and fentanyl, and received lidocaine 10% local anesthesia spray before the transesophageal echocardiography investigation that enabled to visualize better the mass dimensions and where it was located; in the posterior annulus (adjacent to P2 segment of posterior cusp), however it did not add information with regarding the nature and aspect of the mass. Figure 2 and 3 show the medium mass on its largest diameters, 1.8cm x 2.1cm. Atria and appendages did not present thrombus, and the left atrial appendage appeared slightly hypocontractile (dissection speed of 0.34m/s).

The patient underwent a nuclear magnetic resonance of the heart that revealed a rounded image with low magnetic signal at the posterior mitral valve annulus compatible with coarse calcification. Although no anatomopathologic study was performed, echocardiographic findings and the nuclear magnetic resonance suggested, based on evidence of the literature, a diagnosis of caseous calcification of the mitral annulus^(1,3)^.

## DISCUSSION

Caseous calcification of the mitral annulus is rare and accounts for 0.5 to 1% of all calcifications of the mitral annulus. The largest serie in the literature has only 18 cases^(1,2,4)^. It is often represented as an intracardiac mass easily misdiagnosed as tumors or thrombus, which leads to unnecessary surgical interventions. This affection is commonly found in the posterior annulus, medium portions and posterior basal cusp. The more affected population by the disease are women older than 70 years^(4)^.

The anatomopathologic aspect described in the literature is a periannular calcification composed by calcium, fatty acids and cholesterol. Microscopically its interior content has a typical aspect of “toothpaste”, and the prevalence in necropsies corresponds to 2.7% of calcifications of the mitral annulus^(3)^.

In general patients are asymptomatic. However, when symptoms are presented, dyspnea secondary to mitral insufficiency or to mitral stenosis is the most frequent clinical finding. Embolic phenomena, which are rare, might also occur. In addition, there is an association between this type of calcification with hypertension, coronary artery disease and aortic atheromatous disease^(5)^.

The transthoracic echocardiography study is often enough to diagnose the disease, and in most of cases the transesophageal becomes a complement to support the diagnosis. The featured echocardiography image is an echodense mass, rounded and sometimes semilunar with an echolucent area on its interior that is located in the posterior mitral valve annulus^(1)^. The use of transesophageal study enables to define better the local, consistence and aspect of the mass mainly in patients with limited acoustic window. It is important to identify the possibility of this disease and to differentiate it from tumors, thrombus or abscesses^(6)^. In addition, the benign evolution of the disease, peripheral calcification, well-defined edges and its typical localization on the posterior annulus help to differentiate it from abscesses (often found in the mitral-aortic intervalvular fibrosa). In tumors the central echolucent seen in cases of caseous calcification is not observed^(6-8)^.

To recognize and differentiate this disease is crucial to avoid unnecessary surgical interventions, especially because caseous calcification presents a benign evolution and good long-term prognosis. Surgical treatment should be restricted to cases of valves lesions (stenosis or insufficiency) with significant repercussion^(8)^.

We reported a case of a woman with a mass found in mitral valve annulus in which was observed the probable etiology of caseous calcification of the annulus.

## References

[1] Fernandes RM, Branco ML, Galrinho A, Timóteo AT, Tavares A, Feliciano J (2007). Degenerescência caseosa da calcificação do anel mitral: revisão a propósito de 6 casos. Rev Port Cardiol.

[2] Davidson MJ, Cohn LH (2006). Surgical treatment of caseous mitral valve annulus calcific ation. J Thorac Cardiovasc Surg.

[3] Alkadhi H, Leschka S, Prêtre R, Perren A, Marincek B, Wildermuth S (2005). Caseous calcification of the mitral annulus. J Thorac Cardiovasc Surg.

[4] Harpaz D, Auerbach I, Vered Z, Motro M, Tobar A, Rosenblatt S (2001). Caseous calcification of the mitral annulus: a neglected, unrecognized diagnosis. J Am Soc Echocardiogr.

[5] Pomerance A (1970). Pathological and clinical study of calcification of the mitral valve ring. J Clin Pathol.

[6] Deluca G, Correale M, Ieva R, Del Salvatore B, Gramenzi S, Di Biase M (2008). The incidence and clinical course of caseous calcification of the mitral annulus: a prospective echocardiographic study. J Am Soc Echocardiogr.

[7] Gramenzi S, Mazzola AA, Tagliaferri B, Protasoni G, Brusoni D, d'Aloya G (2005). Caseous calcification of the mitral annulus: unusual case of spontaneous resolution. Echocardiography.

[8] García-Ibarrondo N, Lang RM (2011). Caseous calcification of the mitral annulus, a rare echocardiographic finding. Rev Esp Cardiol.

